# Vaccine hesitancy among Saudi parents of children in Abha City, Saudi Arabia: a cross-sectional study

**DOI:** 10.3389/fpubh.2026.1902040

**Published:** 2026-07-27

**Authors:** Afnan Mohammad Aseeri, Abdulaziz Khaled AlZailaie, Naif Saud Abdullah, Ahmad Hasan Alzzaqani, Sultan Abdullah Albqami

**Affiliations:** 1 Preventive Medicine Residency Program, Aseer Health Cluster, Abha, Saudi Arabia; 2 Assistant Professor of Neurosurgery, Department of Surgery, College of Medicine, Najran University, Najran, Saudi Arabia; 3 Saudi Public Health Authority, Riyadh, Saudi Arabia; 4 Aseer Health Cluster, Abha, Saudi Arabia

**Keywords:** childhood vaccination, immunization, parents, Saudi Arabia, vaccine hesitancy

## Abstract

**Background:**

Vaccine hesitancy remains an important global public health concern despite the success of childhood immunization programs. Parental concerns regarding vaccine safety, side effects, and misinformation may negatively influence vaccine uptake and contribute to delayed or refused childhood vaccination. This study aimed to assess vaccine hesitancy among Saudi parents in Abha City and identify factors associated with hesitant attitudes toward compulsory childhood vaccination.

**Methods:**

A cross-sectional analytical study was conducted among 382 Saudi parents of children younger than 12 years in Abha City, Saudi Arabia, between 1 January and 30 April 2025. Data were collected using a structured Arabic questionnaire assessing sociodemographic characteristics, vaccine knowledge, attitudes, trust, and vaccination behaviors. A composite vaccine hesitancy score was calculated, and participants with scores ≥11 were classified as having high vaccine hesitancy. Firth penalized logistic regression analysis was performed to identify factors independently associated with high vaccine hesitancy. Data were analyzed using R statistical software version 4.4.3.

**Results:**

Most participants demonstrated good awareness regarding compulsory childhood vaccination, with 96.9% reporting familiarity with the national immunization schedule. Although overall vaccine confidence was high, 46.1% of participants reported concerns regarding vaccine side effects, and 35.6% had previously delayed or refused a compulsory vaccine for their child. Overall, 5.2% of participants demonstrated high vaccine hesitancy. Firth penalized logistic regression analysis showed that parents older than 40 years (adjusted OR = 6.62, 95% CI: 1.65–38.89, *p* = 0.006) and female participants (adjusted OR = 3.41, 95% CI: 1.29–9.98, *p* = 0.013) were independently associated with high vaccine hesitancy.

**Conclusion:**

Although vaccine hesitancy was relatively low among Saudi parents in Abha City, concerns regarding vaccine safety and side effects remain common. Targeted educational interventions, effective healthcare communication, and strategies addressing vaccine misinformation are essential to maintain public confidence in childhood immunization programs.

## 1 Introduction

Vaccination is considered one of the most effective public health interventions for preventing infectious diseases and reducing childhood morbidity and mortality worldwide. According to the World Health Organization (WHO), immunization currently prevents approximately 3.5 to 5 million deaths annually from diseases such as measles, diphtheria, tetanus, pertussis, and influenza (1). Despite the substantial success of vaccination programs, vaccine hesitancy has emerged as an increasing global public health concern and was recognized by the WHO as a major threat to global health and remains a significant challenge to immunization programs worldwide (2).

Vaccine hesitancy refers to delayed acceptance or refusal of vaccines despite vaccine availability and accessibility (3). Hesitancy is a complex and multifactorial phenomenon influenced by confidence in vaccine safety and effectiveness, complacency toward vaccine-preventable diseases, and convenience of vaccine access (3). Additional contributing factors include misinformation, fear of adverse effects, cultural beliefs, distrust in healthcare systems, and exposure to anti-vaccine content on social media platforms (4, 5).

Globally, vaccine hesitancy has contributed to outbreaks of previously controlled infectious diseases. For example, measles cases increased by approximately 79% worldwide in 2022 compared with the previous year, partly due to declining vaccination coverage in several countries (4). Childhood vaccination coverage also declined during and after the COVID-19 pandemic, increasing concerns regarding the resurgence of vaccine-preventable diseases (6).

In Saudi Arabia, the Ministry of Health has implemented a comprehensive national immunization program providing compulsory childhood vaccines free of charge through primary healthcare centers. The country has generally maintained high childhood vaccination coverage rates exceeding 90% for several routine vaccines (7). However, recent studies from different regions of Saudi Arabia have reported varying levels of parental vaccine hesitancy, delayed childhood vaccination, and concerns regarding vaccine safety despite widespread vaccine availability and high national vaccination coverage (7, 8). Concerns regarding vaccine safety, side effects, and misinformation remain among the most commonly reported factors influencing parental vaccination decisions (4, 8, 9).

Understanding parental attitudes toward compulsory childhood vaccination is particularly important in Saudi Arabia because of the country’s large annual population movements associated with Hajj and Umrah pilgrimages, which may increase the risk of infectious disease transmission. Although national childhood vaccination coverage remains high, vaccine hesitancy continues to represent a potential threat to sustained immunization success. Even relatively low levels of vaccine hesitancy may adversely affect timely vaccine uptake and contribute to delays or selective refusal of childhood immunizations. Furthermore, evidence regarding parental vaccine hesitancy in the Aseer Region remains limited compared with other regions of Saudi Arabia, creating an important gap in understanding regional determinants of vaccine acceptance. Therefore, the present study aimed to assess vaccine hesitancy among Saudi parents in Abha City and identify factors associated with hesitant attitudes toward compulsory childhood vaccination.

## 2 Methods

### 2.1 Study design and setting

A cross-sectional analytical study was conducted among Saudi parents in Abha City, Aseer Region, southwestern Saudi Arabia. Abha is one of the major urban centers in the southern region of the country and is served by an extensive primary healthcare network under the Saudi Ministry of Health. The study was carried out between 1 January and 30 April 2025, with the aim of assessing vaccine hesitancy toward compulsory childhood vaccination and identifying factors associated with hesitant attitudes among parents of children younger than 12 years.

### 2.2 Study population

The target population included Saudi parents residing in Abha City who had at least one child younger than 12 years of age. Both fathers and mothers were eligible to participate in the study. Participants were recruited from the community and primary healthcare settings during the study period.

### 2.3 Inclusion and exclusion criteria

Parents were included if they were Saudi nationals, aged 18 years or older, resided in Abha City, and had at least one child younger than 12 years. Participants were also required to provide informed consent before enrollment in the study.

Parents who did not have children younger than 12 years, non-Saudi residents, individuals unable to complete the questionnaire, and participants with incomplete responses were excluded from the study.

### 2.4 Sample size calculation

The minimum required sample size was calculated using the single population proportion equation for cross-sectional studies:


$$n=Z^{2}P(1‐P)/d^{2}$$

Assuming a 95% confidence level, a margin of error of 5%, and an anticipated prevalence of vaccine hesitancy of 50% to maximize the sample size in the absence of precise local estimates, the calculated minimum sample size was 384 participants. The final analyzed sample included 382 participants with complete responses, which closely approximated the calculated minimum sample size and was considered sufficient for exploratory analysis.

### 2.5 Sampling technique

A non-probability convenience sampling technique was used to recruit eligible participants during the study period. Parents attending selected primary healthcare centers and community settings in Abha City were approached and invited to participate. The purpose of the study was explained to all participants before questionnaire administration.

### 2.6 Study tools

Data were collected using a structured self-administered questionnaire developed after reviewing the World Health Organization Strategic Advisory Group of Experts (WHO SAGE) Vaccine Hesitancy framework and survey tool proposed by Larson et al. The questionnaire incorporated key domains recommended for assessing vaccine hesitancy, including vaccine knowledge, attitudes and beliefs, trust in healthcare providers, social and cultural influences, and vaccination behaviors (3, 4, 7). Although validated Arabic versions of the Parent Attitudes about Childhood Vaccines (PACV) questionnaire are available, the present study aimed to assess a broader range of determinants, including vaccine knowledge, social influences, and future vaccination intentions, which are not comprehensively captured within a single PACV instrument. Therefore, a WHO SAGE-informed multidimensional questionnaire was developed for the study. The final questionnaire consisted of five sections: (1) sociodemographic characteristics, (2) knowledge regarding childhood vaccination, (3) attitudes and beliefs toward compulsory childhood vaccination, (4) social and cultural influences affecting vaccination decisions, and (5) vaccination behaviors and future vaccination intentions.

The questionnaire was originally developed in English and translated into Arabic by bilingual public health researchers. The Arabic version was subsequently reviewed by preventive medicine specialists to assess clarity, cultural appropriateness, and content relevance. A pilot study involving 15 Saudi parents was conducted to evaluate questionnaire comprehensibility, wording, and completion time. Minor linguistic modifications were made based on participant feedback. Data obtained during the pilot phase were not included in the final analysis. The complete Arabic and English versions of the questionnaire are provided in Supplementary Material S1.

### 2.7 Vaccine hesitancy score and classification

A composite vaccine hesitancy score was calculated using eight questionnaire items adapted from the WHO SAGE vaccine hesitancy framework and the study questionnaire (Supplementary Material S1). The items included: C1 (concern about vaccine side effects), C2 (trust in vaccine safety and effectiveness; reverse coded), C3 (belief that compulsory vaccines are administered too early), C4 (belief that natural immunity is preferable to vaccine-acquired immunity), C5 (concern regarding administration of multiple vaccines at one time), D1 (perceived concern about childhood vaccination among family and community members), E1 (previous delay or refusal of a compulsory vaccine), and E3 (concern about giving compulsory vaccines to one’s child). Items assessing knowledge (Section B), family support (D2), religious or cultural beliefs (D3), future vaccination intention (E2), the main reason for concern (E4), and healthcare provider suggestions (E5) were not included in the composite hesitancy score.

Responses indicating greater vaccine hesitancy were assigned higher scores, whereas positively worded items reflecting greater vaccine confidence (e.g., trust in vaccine safety) were reverse coded. For ordinal items, responses were coded as 0 (least hesitant), 1 (intermediate), and 2 (most hesitant). Dichotomous items were coded as 0 (absence of vaccine-hesitant behavior or concern) and 1 (presence of vaccine-hesitant behavior or concern). Individual item scores were summed to generate the composite vaccine hesitancy score, with higher scores indicating greater vaccine hesitancy.

In the study population, observed vaccine hesitancy scores ranged from 3 to 14. Participants with scores ≥11 were classified as having high vaccine hesitancy, whereas those scoring <11 were classified as having low vaccine hesitancy. The ≥11 threshold was predefined during questionnaire development to identify participants demonstrating consistently vaccine-hesitant attitudes across multiple domains rather than isolated concerns or single hesitant responses. The threshold was retained throughout the analysis and was not data-driven or selected *post hoc*.

### 2.8 Reliability assessment

The internal consistency of the vaccine hesitancy scale was assessed using Cronbach’s alpha coefficient. Reliability analysis was initially performed on the nine questionnaire items considered for inclusion in the composite vaccine hesitancy score. Item-total correlation analysis identified the social influence item (D1: “Most people I know are concerned about childhood vaccination”) as having a negative corrected item-total correlation, indicating poor consistency with the remaining vaccine hesitancy items. Consequently, this item was excluded from the final composite score. Removal of D1 improved Cronbach’s alpha from 0.54 to 0.63, indicating modest internal consistency, which was considered acceptable for an exploratory multidimensional instrument.

Content validity was supported through adaptation of questionnaire items from the WHO Strategic Advisory Group of Experts (WHO SAGE) Vaccine Hesitancy framework and previously published vaccine hesitancy questionnaires, followed by expert review and pilot testing. Because the questionnaire was developed as a multidimensional instrument for this study rather than as a formal psychometric scale, exploratory or confirmatory factor analysis was not performed. The complete scoring framework, including the questionnaire items considered for scoring, response coding, identification of the excluded item, and the final scoring algorithm used to calculate the composite vaccine hesitancy score, is presented in Supplementary Table S2 (Supplementary Material 2).

### 2.9 Study variables

The primary outcome variable was vaccine hesitancy grade, categorized as low or high hesitancy based on the composite hesitancy score derived from questionnaire responses.

Independent variables included sociodemographic characteristics such as age, gender, education level, employment status, monthly income, number of children, age of the youngest child, and sex of the youngest child. Additional independent variables included vaccine knowledge, trust in healthcare workers, concerns regarding vaccine side effects, beliefs about vaccine timing and natural immunity, concerns regarding multiple vaccine administration, previous vaccine delay or refusal, and willingness to receive future compulsory vaccines.

Composite scores for vaccine knowledge, attitudes, trust, and vaccination behaviors were generated using coded questionnaire responses and were used for descriptive analyses of vaccine hesitancy-related domains.

### 2.10 Statistical analysis

Data were entered, cleaned, and analyzed using R statistical software version 4.4.3 (R Foundation for Statistical Computing, Vienna, Austria). Descriptive statistics were used to summarize participant characteristics and questionnaire responses. Categorical variables were presented as frequencies and percentages, whereas continuous variables were summarized using means and standard deviations. Bivariate analyses were performed to assess associations between participant characteristics and vaccine hesitancy grade using Pearson’s chi-square test or Fisher’s exact test, as appropriate. To identify factors independently associated with high vaccine hesitancy, Firth penalized logistic regression was performed. This approach was selected because only a small proportion of participants were classified as highly hesitant, resulting in sparse outcome events and potential separation issues. Adjusted odds ratios (ORs) and corresponding 95% confidence intervals (CIs) were calculated from the penalized regression model.

As a sensitivity analysis, multivariable linear regression was additionally performed using the continuous vaccine hesitancy score as the outcome variable. Reliability analysis of the hesitancy-related items was performed using Cronbach’s alpha coefficient. Composite hesitancy-related scores were additionally analyzed as continuous variables to improve statistical stability and preserve information variability. Statistical significance was defined as a two-sided *p*-value < 0.05.

### 2.11 Ethical considerations

The study protocol was reviewed and approved by the Institutional Review Board of Aseer Health Cluster, Saudi Arabia (Approval No. IRB-132-2025). Participation was voluntary, and electronic informed consent was obtained from all participants before questionnaire completion. Confidentiality and anonymity of participant information were maintained throughout the study, and all procedures were conducted in accordance with the ethical principles of the Declaration of Helsinki.

## 3 Results

### 3.1 Sociodemographic characteristics and vaccine knowledge

A total of 382 Saudi parents residing in Abha City participated in the study. More than half of the participants were aged 30–40 years (56.5%), while 23.0% were younger than 30 years and 20.4% were older than 40 years (Table 1). Male participants represented 52.9% of the sample. Most participants had a university-level education (74.6%), whereas only 1.6 and 2.1% had primary and intermediate education levels, respectively. Half of the participants were employed in non-healthcare sectors (50.0%), while 19.1% worked in healthcare-related fields and 30.9% were unemployed.

**Table 1:** Sociodemographic characteristics and vaccine knowledge of Saudi parents in Abha City (*n* = 382).

Characteristic	Category	*n* (%)
Age	< 30 years	88 (23.0)
	30–40 years	216 (56.5)
	> 40 years	78 (20.4)
Gender	Male	202 (52.9)
	Female	180 (47.1)
Education level	Primary	6 (1.6)
	Intermediate	8 (2.1)
	Secondary	83 (21.7)
	University	285 (74.6)
Employment status	Unemployed	118 (30.9)
	Healthcare-related	73 (19.1)
	Non-healthcare	191 (50.0)
Monthly income	<10,000 SR	219 (57.3)
	10,000–20,000 SR	149 (39.0)
	>20,000 SR	14 (3.7)
Number of children	One	122 (31.9)
	2–4	210 (55.0)
	>4	50 (13.1)
Age of youngest child	<1 year	189 (49.5)
	1–2 years	111 (29.1)
	3–5 years	42 (11.0)
	>5 years	40 (10.5)
Sex of youngest child	Male	200 (52.4)
	Female	182 (47.6)
Vaccine knowledge	Familiar with compulsory vaccination schedule	370 (96.9)
	Vaccines protect against serious diseases	344 (90.1)
	Vaccines are pre-tested for safety	318 (83.2)

SR, Saudi Riyal.

Regarding monthly household income, 57.3% reported earning less than 10,000 Saudi Riyals (SR), and 39.0% reported incomes between 10,000 and 20,000 SR. Most participants had between two and four children (55.0%), and approximately half reported that their youngest child was younger than 1 year of age (49.5%). Male children represented 52.4% of the youngest children reported by participants. Concerning vaccine knowledge, the majority of participants were familiar with the compulsory vaccination schedule in Saudi Arabia (96.9%), recognized that vaccines protect children against serious diseases (90.1%), and believed that vaccines are pre-tested for safety before administration (83.2%).

### 3.2 Attitudes, beliefs, social influences, and vaccination behaviors

**Figure 1:**
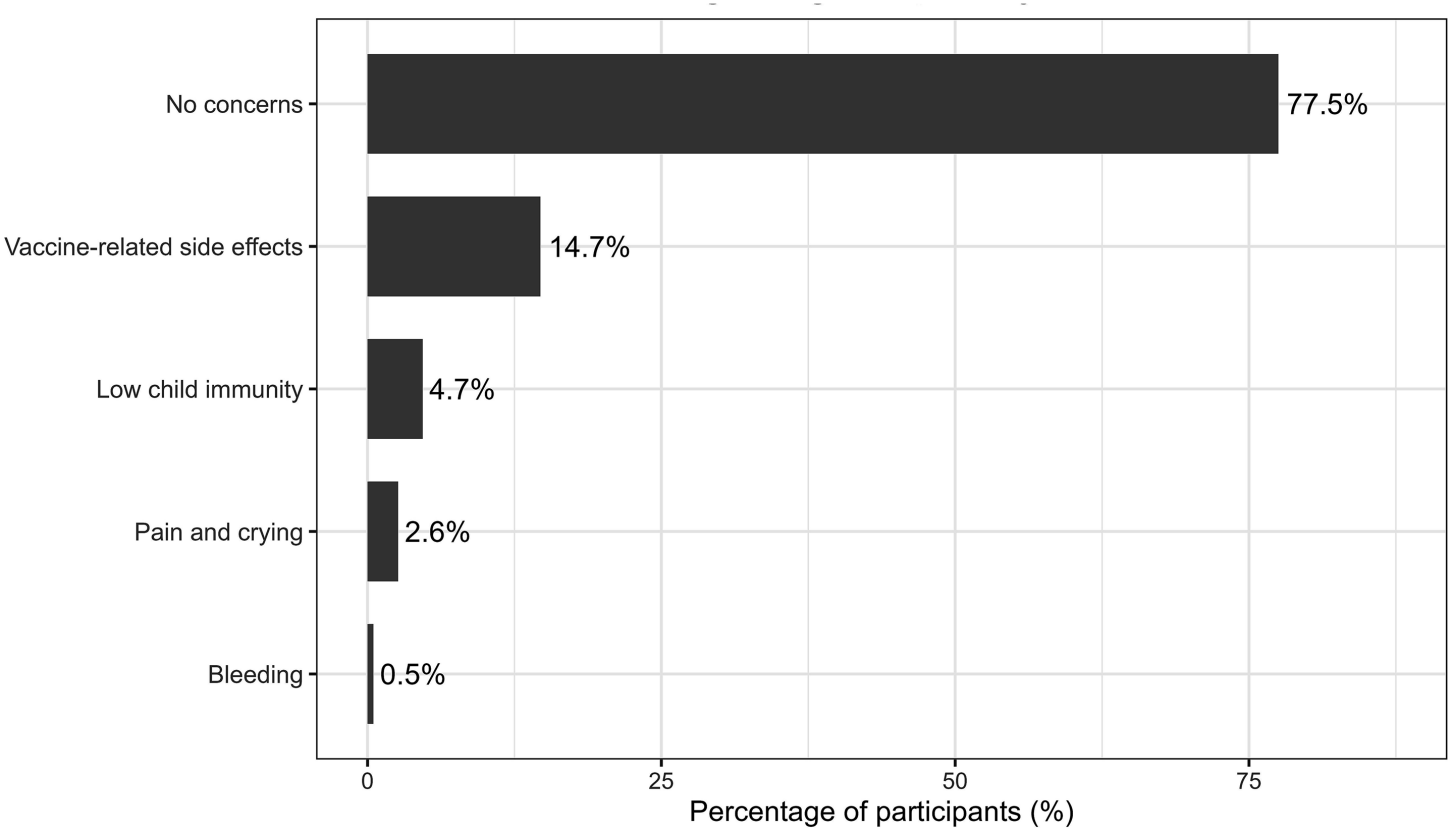
Main parental concerns regarding compulsory childhood vaccination among Saudi parents in Abha City. Vaccine-related side effects were the most commonly reported parental concern regarding compulsory childhood vaccination, followed by concerns related to low child immunity, pain and crying, and bleeding after vaccination. Percentages represent the proportion of all participants. The category “No concerns” (77.5%) is displayed to provide the full distribution of responses.

Nearly half of the participants (46.1%) reported sometimes being concerned about vaccine side effects, while 21.5% were always concerned. Most participants expressed strong trust in healthcare workers regarding vaccine safety and effectiveness (71.7%). Concerns regarding early vaccine administration and receiving multiple vaccines simultaneously were reported by 79.6 and 46.3% of participants, respectively. More than one-third of parents (35.6%) reported previously delaying or refusing a compulsory vaccine. However, among participants who responded to the future vaccination question (*n* = 334), 95.2% indicated that they would allow their children to receive all compulsory vaccines in the future. Overall, 77.5% of participants reported no concerns regarding compulsory childhood vaccination. Among those reporting concerns, vaccine-related side effects were the most frequently cited concern (14.7%), followed by low child immunity (4.7%), pain and crying after vaccination (2.6%), and bleeding (0.5%) (Table 2; Figure 1). Although 35.6% of participants reported previous vaccine delay or refusal, only 5.2% met the predefined criteria for high vaccine hesitancy based on the composite score.

**Table 2:** Attitudes, beliefs, social influences, and vaccination behaviors regarding compulsory childhood vaccination among Saudi parents in Abha City (*n* = 382).

Variable	Category	*n* (%)
Concern about possible vaccine side effects	Never	124 (32.5)
	Sometimes	176 (46.1)
	Always	82 (21.5)
Trust in healthcare workers regarding vaccine safety and effectiveness	Do not trust	6 (1.6)
	To some extent	102 (26.7)
	To a large extent	274 (71.7)
Belief that compulsory vaccines are given too early in life	Agree	304 (79.6)
	Neutral	58 (15.2)
	Disagree	20 (5.2)
Belief that natural immunity is better than vaccine-acquired immunity	Agree	86 (22.5)
	Neutral	158 (41.4)
	Disagree	138 (36.1)
Concern about receiving multiple vaccines at one time	Agree	177 (46.3)
	Neutral	133 (34.8)
	Disagree	72 (18.8)
Family and friends support compulsory vaccination	Yes	272 (71.2)
	No	110 (28.8)
Religious or cultural beliefs negatively affect vaccination decisions	Yes	80 (20.9)
	No	302 (79.1)
Ever delayed or refused a compulsory vaccine for a child	Yes	136 (35.6)
	No	246 (64.4)
Willingness to allow children to receive all compulsory vaccines in the future	Yes	318 (95.2)*
	No	16 (4.8)*
Concerned about giving compulsory vaccines to children	Yes	86 (22.5)
	No	296 (77.5)
Main concerns regarding compulsory childhood vaccination	No concerns	296 (77.5)
	Vaccine-related side effects	56 (14.7)
	Low child immunity	18 (4.7)
	Pain and crying	10 (2.6)
	Bleeding	2 (0.5)

Values are presented as frequency (percentage). *Percentages were calculated using valid responses only (*n* = 334); 48 participants (12.6%) did not respond to this question. SR, Saudi Riyal.

### 3.3 Vaccine hesitancy score and reliability analysis

The mean vaccine hesitancy score among participants was 7.01 ± 2.30, with observed scores ranging from 3 to 14 (Table 3). Based on the predefined classification threshold, participants with scores of 11 or greater were categorized as having high vaccine hesitancy. Reliability analysis demonstrated moderate internal consistency of the original hesitancy scale (Cronbach’s *α* = 0.54). Following removal of one negatively correlated item during item refinement, the internal consistency improved to Cronbach’s α = 0.63, indicating acceptable reliability for exploratory research purposes.

**Table 3:** Vaccine hesitancy score distribution and reliability analysis among Saudi parents in Abha City (*n* = 382).

Variable	Mean ± SD	Observed score range
Total vaccine hesitancy score	7.01 ± 2.30	3–14

SD, Standard deviation. The composite vaccine hesitancy score was derived by summing the scores of hesitancy-related questionnaire items, with higher scores indicating greater vaccine hesitancy. Observed scores ranged from 3 to 14. Participants with scores <11 were classified as having low vaccine hesitancy, whereas those with scores ≥11 were classified as having high vaccine hesitancy. The internal consistency reliability of the vaccine hesitancy scale was assessed using Cronbach’s alpha. The initial scale demonstrated moderate reliability (Cronbach’s *α* = 0.54). After removal of one negatively correlated item during item refinement, the final scale showed improved internal consistency (Cronbach’s α = 0.63).

### 3.4 Comparison according to vaccine hesitancy grade

Overall, 20 participants (5.2%) demonstrated high vaccine hesitancy. Significant associations were observed between vaccine hesitancy and age, gender, employment status, and sex of the youngest child. Parents older than 40 years and female participants were more likely to exhibit high vaccine hesitancy (*p* < 0.001 and *p* = 0.035, respectively). High hesitancy was also more common among non-healthcare workers (*p* = 0.008) and among parents whose youngest child was male (*p* = 0.011). No significant associations were found for education level, monthly income, number of children, or age of the youngest child (Table 4).

**Table 4:** Comparison of sociodemographic characteristics according to vaccine hesitancy grade among Saudi parents in Abha City.

Variable	Category	Low hesitancy (*n* = 362)	High hesitancy (*n* = 20)	*p*-value
Age	<30 years	86 (23.8)	2 (10.0)	<0.001*
	30–40 years	212 (58.6)	4 (20.0)	
	>40 years	64 (17.7)	14 (70.0)	
Gender	Male	196 (54.1)	6 (30.0)	0.035*
	Female	166 (45.9)	14 (70.0)	
Education level	Primary	6 (1.7)	0 (0.0)	0.990
	Intermediate	8 (2.2)	0 (0.0)	
	Secondary	79 (21.8)	4 (20.0)	
	University	269 (74.3)	16 (80.0)	
Employment status	Unemployed	114 (31.5)	4 (20.0)	0.008*
	Healthcare-related	73 (20.2)	0 (0.0)	
	Non-healthcare	175 (48.3)	16 (80.0)	
Monthly income	<10,000 SR	211 (58.3)	8 (40.0)	0.200
	10,000–20,000 SR	137 (37.8)	12 (60.0)	
	>20,000 SR	14 (3.9)	0 (0.0)	
Number of children	One	118 (32.6)	4 (20.0)	0.400
	2–4	196 (54.1)	14 (70.0)	
	>4	48 (13.3)	2 (10.0)	
Age of youngest child	<1 year	183 (50.6)	6 (30.0)	0.200
	1–2 years	103 (28.5)	8 (40.0)	
	3–5 years	38 (10.5)	4 (20.0)	
	>5 years	38 (10.5)	2 (10.0)	
Sex of youngest child	Male	184 (50.8)	16 (80.0)	0.011*
	Female	178 (49.2)	4 (20.0)	

Data are presented as *n* (%). * Statistically significant at *p* < 0.05. *p*-values were calculated using Pearson’s chi-square test. Fisher’s exact test was applied when expected cell counts were <5.

### 3.5 Factors associated with high vaccine hesitancy

To account for the small number of participants classified as highly vaccine hesitant (n = 20) and to address sparse-data separation, Firth penalized logistic regression was performed. As shown in Table 5, parental age and gender were independently associated with high vaccine hesitancy. Parents older than 40 years had significantly greater odds of exhibiting high vaccine hesitancy compared with those younger than 30 years (adjusted OR = 6.62, 95% CI: 1.65–38.89, *p* = 0.006). Similarly, female participants were more likely to demonstrate high vaccine hesitancy than male participants (adjusted OR = 3.41, 95% CI: 1.29–9.98, *p* = 0.013). No significant associations were observed for employment status. Although healthcare-related employment showed a tendency toward lower vaccine hesitancy (adjusted OR = 0.19, 95% CI: 0.001–1.93), this association did not reach statistical significance (*p* = 0.188). Likewise, non-healthcare employment was not significantly associated with high vaccine hesitancy compared with unemployment (adjusted OR = 1.60, 95% CI: 0.47–6.23, *p* = 0.456).

**Table 5:** Firth penalized logistic regression analysis of factors associated with high vaccine hesitancy among saudi parents in Abha City (*n* = 382).

Variable	Category	Adjusted OR	95% CI	*p*-value
Age	<30 years	Reference	—	—
	30–40 years	0.80	0.17–4.71	0.782
	>40 years	6.62	1.65–38.89	0.006*
Gender	Male	Reference	—	—
	Female	3.41	1.29–9.98	0.013*
Employment status	Unemployed	Reference	—	—
	Healthcare-related	0.19	0.001–1.93	0.188
	Non-healthcare	1.60	0.47–6.23	0.456

OR, Odds Ratio; CI, Confidence Interval. Estimates were obtained using Firth penalized logistic regression to reduce small-sample bias and address sparse-data separation arising from the low number of participants with high vaccine hesitancy (*n* = 20). Odds ratios were calculated by exponentiating regression coefficients. *p*-values were derived using profile penalized likelihood methods. *Statistically significant at *p* < 0.05.

## 4 Discussion

The present study explored vaccine hesitancy among Saudi parents of children younger than 12 years in Abha City and identified factors associated with hesitancy toward compulsory childhood vaccination. Although overall vaccine confidence was high, a measurable proportion of parents expressed concerns regarding vaccine safety, side effects, and administration of multiple vaccines simultaneously. Only 5.2% of participants met the predefined threshold for high vaccine hesitancy based on the composite vaccine hesitancy score, although concerns regarding vaccination remained common. These findings suggest that while confidence in childhood vaccination is generally strong, specific misconceptions and safety concerns persist. Similar observations have been reported in regional and international studies demonstrating that vaccine hesitancy remains an important public health challenge despite widespread immunization programs (4, 10–12).

Most participants demonstrated good knowledge regarding compulsory childhood vaccination. Nearly all participants were familiar with the Saudi vaccination schedule and recognized the protective role and safety testing of vaccines. Similar findings have been reported in other Saudi studies, reflecting the effectiveness of national immunization programs and public awareness initiatives (7). Nevertheless, the persistence of hesitancy despite high knowledge levels suggests that vaccine hesitancy is influenced not only by knowledge deficits but also by emotional, psychological, and social factors, including mistrust, fear of adverse effects, and misinformation exposure (3, 4). Accordingly, assessment of vaccine hesitancy should extend beyond factual knowledge to include confidence, beliefs, social influences, and vaccination behaviors, supporting the multidimensional approach adopted in the present study.

Concerns regarding vaccine side effects represented the most frequently reported parental concern in the current study. Approximately one-fifth of participants reported always being concerned about vaccine side effects, while nearly half were sometimes concerned. Fear of adverse events has consistently been identified as one of the strongest contributors to vaccine hesitancy worldwide (5, 13). Similarly, concerns regarding administration of multiple vaccines simultaneously were relatively common among participants. Previous literature has shown that some parents perceive multiple vaccines as potentially overwhelming the child’s immune system despite substantial scientific evidence supporting the safety and effectiveness of routine vaccination schedules (4, 14).

Although most participants reported trusting healthcare workers regarding vaccine safety and effectiveness, more than one-third had previously delayed or refused a compulsory vaccine for their child. At first glance, this appears inconsistent with the relatively low prevalence of high vaccine hesitancy identified in the present study (5.2%). However, vaccine delay or refusal may occur for reasons other than persistent vaccine hesitancy, including temporary concerns regarding child illness, scheduling difficulties, access-related barriers, or precautionary delays recommended by healthcare providers. In contrast, the composite hesitancy score was designed to identify parents with consistently hesitant attitudes across multiple domains, including confidence, beliefs, concerns, and vaccination behaviors. Similar discrepancies between vaccination behavior and attitudinal measures of hesitancy have been reported in previous vaccine hesitancy research (5). Therefore, previous vaccine delay or refusal should not be interpreted as synonymous with persistent vaccine hesitancy, highlighting the importance of multidimensional assessment rather than reliance on a single behavioral indicator.

Social support for vaccination was generally high, with most participants reporting positive support from family members and friends. In contrast, religious and cultural beliefs negatively influenced vaccination decisions among only a minority of participants. These findings likely reflect the generally favorable societal and religious attitudes toward vaccination in Saudi Arabia. Previous evidence suggests that endorsement of vaccination by healthcare professionals, governmental agencies, and religious leaders can enhance public confidence and reduce vaccine-related misconceptions (15). Nevertheless, culturally sensitive vaccine education remains important to address persistent misconceptions, strengthen public trust, and ensure continued acceptance of childhood immunization programs. Interventions should address not only knowledge gaps but also concerns related to vaccine safety, confidence in healthcare providers, and social influences that may affect parental vaccination decisions, particularly among groups expressing vaccine-related concerns (16).

The present study demonstrated a relatively low prevalence of high vaccine hesitancy, with only 20 participants (5.2%) exceeding the predefined composite score threshold (≥11). This finding suggests generally favorable attitudes toward compulsory childhood vaccination among Saudi parents in Abha City. The prevalence observed in the current study appears lower than that reported in some Western populations where vaccine skepticism and anti-vaccine movements are more prominent, but is broadly consistent with findings from several Gulf countries characterized by strong public trust in governmental healthcare systems (8). Nevertheless, even low levels of hesitancy remain important because hesitant parents may contribute disproportionately to delayed immunization, selective vaccine refusal, and reduced acceptance of future vaccination campaigns (2).

One of the principal findings of the present study was that parents older than 40 years were significantly more likely to exhibit high vaccine hesitancy than younger parents. This association remained significant after adjustment for other sociodemographic variables. Although previous studies have reported inconsistent findings regarding the relationship between parental age and vaccine hesitancy, older age has been associated with greater reliance on established beliefs, prior experiences, and alternative information sources that may influence vaccine perceptions (8, 17). The relatively wide confidence interval observed in the current study suggests that the magnitude of this association should be interpreted cautiously, although the direction of the relationship remained consistent.

Female participants demonstrated significantly greater odds of vaccine hesitancy than male participants. This finding is consistent with several previous studies reporting higher levels of vaccine-related concern among mothers and female caregivers (8, 18). Women are often the primary decision-makers regarding childhood healthcare and may therefore be more attentive to potential vaccine risks and adverse effects. Greater engagement with parenting networks and social media discussions may also increase exposure to vaccine-related concerns and misinformation, potentially contributing to heightened hesitancy (19, 20).

Because only a small proportion of participants were classified as highly vaccine hesitant, Firth penalized logistic regression was used to minimize small-sample bias and address sparse-data separation (21). Although this approach improves the stability of estimates compared with conventional logistic regression, the relatively small number of high-hesitancy participants limits the precision of some effect estimates (21, 22). Consequently, the identified associations should be interpreted as exploratory and require confirmation in larger studies.

The findings of the current study have several public health implications. Continuous vaccine education campaigns remain necessary despite generally high vaccine acceptance levels. Educational interventions should particularly address concerns regarding vaccine safety, side effects, and administration of multiple vaccines (16). In addition, healthcare providers should receive training in effective vaccine communication strategies, including empathetic counseling and evidence-based discussions with hesitant parents (13).

Public health authorities should also strengthen digital communication strategies to counter vaccine misinformation on social media platforms, which increasingly influence parental health decisions (23). Community-based interventions involving schools, healthcare providers, and religious leaders may further strengthen vaccine confidence and improve trust in immunization programs (16). Future multicenter studies using standardized and validated vaccine hesitancy instruments alongside broader contextual assessments would facilitate comparisons across different regions of Saudi Arabia and improve understanding of factors influencing parental vaccine acceptance. Finally, ongoing monitoring of vaccine hesitancy trends is important for identifying emerging concerns and supporting future vaccination strategies (3).

### 4.1 Limitations

Several limitations should be considered when interpreting the findings of the present study. First, the cross-sectional design precludes establishing causal relationships between participant characteristics and vaccine hesitancy. Second, participants were recruited using convenience sampling from a single city in the Aseer Region of Saudi Arabia, which may limit the representativeness and generalizability of the findings to other regions or populations. Third, data were collected through self-reported questionnaires and may therefore be subject to recall bias and social desirability bias.

Fourth, only a small proportion of participants (5.2%) were classified as highly vaccine hesitant. Although Firth penalized logistic regression was used to reduce small-sample bias and address sparse-data separation, the limited number of outcome events may have affected the precision of some estimates, as reflected by relatively wide confidence intervals. Consequently, the identified predictors should be interpreted with caution.

Finally, although the vaccine hesitancy questionnaire was developed using domains derived from the WHO SAGE Vaccine Hesitancy framework and demonstrated acceptable internal consistency after item refinement (Cronbach’s *α* = 0.63), formal psychometric validation, including factor analysis and construct validation, was not performed. Therefore, comparisons with studies using established validated instruments should be interpreted cautiously. Future multicenter studies employing validated vaccine hesitancy scales and larger, more representative samples are warranted to confirm and extend the present findings.

## 5 Conclusion

The present study demonstrated generally favorable attitudes toward compulsory childhood vaccination among Saudi parents in Abha City, with only a small proportion of participants classified as highly vaccine hesitant. Nevertheless, concerns regarding vaccine side effects, vaccine safety, and the administration of multiple vaccines remained common among some parents. Older parental age and female gender were independently associated with higher vaccine hesitancy. These findings suggest that vaccine hesitancy in this population is driven more by concerns and perceptions than by a lack of basic vaccine knowledge. Targeted educational initiatives, effective communication by healthcare providers, and evidence-based strategies to address vaccine-related concerns and misinformation may help further strengthen parental confidence in childhood vaccination. Continued monitoring of vaccine hesitancy and parental attitudes is essential to sustain high immunization coverage and support ongoing public health efforts in Saudi Arabia.

## Data Availability

The original contributions presented in the study are included in the article/Supplementary material, further inquiries can be directed to the corresponding author.
