# Supplementary material for: Vaccine hesitancy among Saudi parents of children in Abha City, Saudi Arabia: a cross-sectional study
Source: Front Public Health. 2026 Jul 27;14:1902040. doi: 10.3389/fpubh.2026.1902040 (PMC13454214; doi:10.3389/fpubh.2026.1902040)
Supplement: Supplementary file 1 [file Presentation_1.PDF]

# Supplementary Material S1

## Questionnaire Used in the Study

*Vaccine Hesitancy Among Saudi Parents of Children in Abha City, Saudi Arabia: A Cross-Sectional Study*

## Section A: Sociodemographic Characteristics

| Item Number | Arabic | English and Response Options |
|---|---|---|
| **A1** | العمر | **Age**<br>☐ <30 years<br>☐ 30–40 years<br>☐ >40 years |
| **A2** | الجنس | **Gender**<br>☐ Male<br>☐ Female |
| **A3** | مستوى تعليمك | **Education level**<br>☐ Primary<br>☐ Intermediate<br>☐ Secondary<br>☐ University |
| **A4** | الحالة الوظيفية | **Employment status**<br>☐ Unemployed<br>☐ Healthcare-related<br>☐ Non-healthcare |
| **A5** | متوسط الدخل الشهري للأسرة | **Monthly household income**<br>☐ <10,000 SR<br>☐ 10,000–20,000 SR<br>☐ >20,000 SR |
| **A6** | عدد الأطفال | **Number of children**<br>☐ One<br>☐ 2–4<br>☐ >4 |

| Item Number | Arabic | English and Response Options |
|---|---|---|
| A7 | عمر أصغر طفل لديك | **Age of youngest child**<br>☐ <1 year<br>☐ 1–2 years<br>☐ 3–5 years<br>☐ >5 years |
| A8 | جنس أصغر طفل لديك | **Sex of youngest child**<br>☐ Male<br>☐ Female |

## Section B: Knowledge About Childhood Vaccination

| Item Number | Arabic | English and Response Options |
|---|---|---|
| B1 | ما مدى معرفتك بجدول التطعيمات الإلزامية في المملكة العربية السعودية؟ | **How familiar are you with the compulsory vaccination schedule in Saudi Arabia?**<br>☐ Not familiar<br>☐ Somewhat familiar<br>☐ Very familiar |
| B2 | اللقاحات تحمي الأطفال من الأمراض الخطيرة. | **Vaccines protect children against serious diseases.**<br>☐ Yes<br>☐ No<br>☐ Maybe |

| Item Number | Arabic | English and Response Options |
|---|---|---|
| **B3** | هل يتم اختبار اللقاحات للتأكد من سلامتها قبل استخدامها؟ | **Vaccines are tested before being approved for use.**<br>☐ Yes<br>☐ No<br>☐ Maybe |

## Section C: Attitudes and Beliefs Toward Vaccination

| Item Number | Arabic | English and Response Options |
|---|---|---|
| **C1** | هل تشعر بالقلق عند إعطاء طفلك اللقاحات بسبب الآثار الجانبية المحتملة؟ | **How concerned are you about possible vaccine side effects?**<br>☐ Never<br>☐ Sometimes<br>☐ Always |
| **C2** | أثق في سلامة وفعالية اللقاحات التي تُعطى للأطفال. | **I trust the safety and effectiveness of vaccines given to children.**<br>☐ To a large extent<br>☐ To some extent<br>☐ I do not trust |
| **C3** | أرى أنه من الممكن إعطاء اللقاحات للأطفال في عمر مبكر أكثر من اللازم. | **Compulsory vaccines are given too early in life.**<br>☐ Agree<br>☐ Neutral<br>☐ Disagree |

| Item Number | Arabic | English and Response Options |
|---|---|---|
| **C4** | أرى أن ترك الطفل ليحصل على المناعة الطبيعية أفضل من المناعة المكتسبة من اللقاحات. | **Natural immunity is better than vaccine-acquired immunity.**<br>☐ Agree<br>☐ Neutral<br>☐ Disagree |
| **C5** | لا يجب أن يتم تطعيم طفلي بعدة لقاحات في نفس الوقت. | **I am worried about the number of vaccines my child receives at one time.**<br>☐ Agree<br>☐ Neutral<br>☐ Disagree |

## Section D: Social and Cultural Influences

| Item Number | Arabic | English and Response Options |
|---|---|---|
| **D1** | معظم من أعرفهم يقلقون من إعطاء التطعيمات للأطفال. | **Most people I know are concerned about childhood vaccination.**<br>☐ Agree<br>☐ Neutral<br>☐ Disagree |
| **D2** | يدعم أفراد عائلتي وأصدقائي التطعيمات الإلزامية للأطفال. | **My family and friends support compulsory childhood vaccination.**<br>☐ Yes<br>☐ No |

| Item Number | Arabic | English and Response Options |
|---|---|---|
| D3 | معتقداتي الدينية أو الثقافية تؤثر سلبًا على قراري بشأن التطعيمات الإلزامية. | **My religious or cultural beliefs negatively affect my decision regarding compulsory vaccination.**<br>☐ Yes<br>☐ No |

## Section E: Vaccination Behaviors and Future Intentions

| Item Number | Arabic | English and Response Options |
|---|---|---|
| E1 | هل سبق لك أن أخرت أو رفضت تطعيمًا إلزاميًا لطفلك؟ | **Have you ever delayed or refused a compulsory vaccine for your child?**<br>☐ Yes<br>☐ No |
| E2 | في المستقبل، هل ستسمح لطفلك بتلقي جميع التطعيمات الإلزامية؟ | **In the future, are you willing to allow your child to receive all compulsory vaccines?**<br>☐ Yes<br>☐ No |
| E3 | هل لديك أية مخاوف بشأن تلقي طفلك اللقاحات الإلزامية؟ | **Are you concerned about giving compulsory vaccines to your child?**<br>☐ Yes<br>☐ No |

| Item Number | Arabic | English and Response Options |
|---|---|---|
| **E4** | ما هي أكثر مخاوفك بشأن تلقي طفلك اللقاحات الإلزامية؟ | **What is your main concern regarding compulsory childhood vaccination?**<br>☐ No concerns<br>☐ Vaccine-related side effects<br>☐ Bleeding<br>☐ Pain and crying<br>☐ Low child immunity |
| **E5** | ماذا يمكن لمقدمي الرعاية الصحية فعله لزيادة ثقتك بالتطعيمات الإلزامية للأطفال؟ | **What can healthcare providers do to increase your confidence in compulsory childhood vaccination?**<br>☐ Health education about vaccination schedules<br>☐ Increase public awareness<br>☐ Provision of proper vaccines<br>☐ Provide information about compulsory vaccines |

---

*Items related to vaccine confidence, beliefs, trust, and vaccination behavior were adapted from the WHO Strategic Advisory Group of Experts (SAGE) Vaccine Hesitancy Framework and related vaccine hesitancy assessment tools.*
