# Supplementary material for: Vaccine hesitancy among Saudi parents of children in Abha City, Saudi Arabia: a cross-sectional study
Source: Front Public Health. 2026 Jul 27;14:1902040. doi: 10.3389/fpubh.2026.1902040 (PMC13454214; doi:10.3389/fpubh.2026.1902040)
Supplement: Supplementary file 2 [file Supplementary_file_1.DOCX]

**Supplementary table 1.** Multivariable linear regression analysis of factors associated with vaccine hesitancy score among Saudi parents in Abha City (n = 382)

| **Variable** | **Category** | **β Coefficient** | **95% CI** | **p-value** |
| --- | --- | --- | --- | --- |
| **Age** | <30 years | Reference | — | — |
|  | 30–40 years | 0.21 | - 0.27 to 0.68 | 0.395 |
|  | >40 years | 1.21 | 0.55 to 1.88 | <0.001* |
| **Gender** | Male | Reference | — | — |
|  | Female | 0.79 | 0.32 to 1.26 | <0.001* |
| **Education level** | Primary | Reference | — | — |
|  | Intermediate | - 0.63 | - 2.62 to 1.37 | 0.537 |
|  | Secondary | - 1.04 | - 2.63 to 0.55 | 0.200 |
|  | University | - 0.66 | - 2.23 to 0.91 | 0.410 |
| **Employment status** | Unemployed | Reference | — | — |
|  | Healthcare-related | - 0.72 | - 1.33 to -0.12 | 0.020* |
|  | Non-healthcare | 0.38 | - 0.13 to 0.90 | 0.145 |
| **Monthly income** | <10,000 SR | Reference | — | — |
|  | 10,000–20,000 SR | 0.23 | - 0.17 to 0.63 | 0.266 |
|  | >20,000 SR | - 0.71 | - 1.79 to 0.38 | 0.201 |
| **Number of children** | One | Reference | — | — |
|  | 2–4 | 0.03 | - 0.39 to 0.45 | 0.890 |
|  | >4 | - 0.12 | - 0.75 to 0.51 | 0.703 |
| **Age of youngest child** | <1 year | Reference | — | — |
|  | 1–2 years | 0.11 | - 0.33 to 0.56 | 0.621 |
|  | 3–5 years | -0.14 | -0.82 to 0.55 | 0.696 |
|  | >5 years | -0.90 | - 1.64 to - 0.17 | 0.016* |
| **Sex of youngest child** | Male | Reference | — | — |
|  | Female | - 0.53 | - 0.90 to - 0.16 | 0.006* |

**Abbreviations:** CI = Confidence Interval; β = Unstandardized regression coefficient; SR = Saudi Riyal. **Note:** Multivariable linear regression was performed using the total vaccine hesitancy score as a continuous outcome variable. In the study sample, observed scores ranged from 3 to 14, with higher scores indicating greater vaccine hesitancy. Positive β coefficients indicate higher vaccine hesitancy scores, whereas negative β coefficients indicate lower hesitancy scores. The overall model was statistically significant (F = 4.78, p < 0.001) and explained 17.3% of the variance in vaccine hesitancy scores (R² = 0.173; adjusted R² = 0.137). * Statistically significant at p < 0.05.

**Supplementary table S2.** Scoring framework and coding algorithm used to calculate the composite vaccine hesitancy score

| **Questionnaire section** | **Item No. (Supplementary Material S1)** | **Questionnaire item** | **Response coding for hesitancy score** | **Maximum score** | **Included in final composite score** |
| --- | --- | --- | --- | --- | --- |
| C | C1 | Concern about possible vaccine side effects | Never = 0; Sometimes = 1; Always = 2 | 2 | Yes |
| C | C2 | Trust in healthcare workers regarding vaccine safety and effectiveness | To a large extent = 0; To some extent = 1; Do not trust = 2 | 2 | Yes |
| C | C3 | Belief that compulsory vaccines are given too early in life | Disagree = 0; Neutral = 1; Agree = 2 | 2 | Yes |
| C | C4 | Belief that natural immunity is better than vaccine-acquired immunity | Disagree = 0; Neutral = 1; Agree = 2 | 2 | Yes |
| C | C5 | Concern about receiving multiple vaccines at one time | Disagree = 0; Neutral = 1; Agree = 2 | 2 | Yes |
| D | D1 | Most people I know are concerned about childhood vaccination | Disagree = 0; Neutral = 1; Agree = 2 | 2 | No* |
| D | D2 | Previous delay or refusal of a compulsory childhood vaccine | No = 0; Yes = 1 | 1 | Yes |
| E | E1 | Willingness to allow children to receive all compulsory vaccines in the future | Yes = 0; No = 1 | 1 | Yes |
| E | E2 | Concerned about giving compulsory vaccines to children | No = 0; Yes = 1 | 1 | Yes |

**Note:** The initial scoring framework consisted of nine questionnaire items with a maximum possible score of 16 points. During reliability assessment, item D1 ("Most people I know are concerned about childhood vaccination") demonstrated a negative corrected item-total correlation and was therefore excluded from the final composite score. Removal of this item improved Cronbach's alpha from 0.54 to 0.63, resulting in a final eight-item composite vaccine hesitancy score with a maximum possible score of 14 points. Participants with a total score ≥11 were classified as having high vaccine hesitancy, whereas those scoring <11 were classified as having low vaccine hesitancy.
